# Printed, Flexible Lactate Sensors: Design Considerations Before Performing On-Body Measurements

**DOI:** 10.1038/s41598-019-49689-7

**Published:** 2019-09-23

**Authors:** Margaret E. Payne, Alla Zamarayeva, Veronika I. Pister, Natasha A. D. Yamamoto, Ana Claudia Arias

**Affiliations:** University of California Berkeley, Electrical Engineering and Computer Science, Berkeley, CA 94720 United States

**Keywords:** Biomedical materials, Electrical and electronic engineering, Enzymes, Enzyme mechanisms

## Abstract

This work reports the process of sensor development, optimization, and characterization before the transition to on-body measurements can be made. Sensors using lactate oxidase as a sensing mechanism and tetrathiafulvalene as a mediator were optimized for sporting applications. Optimized sensors show linear range up to 24 mM lactate and sensitivity of 4.8 *μ*A/mM which normalizes to 68 *μ*A*cm^−2^/mM when accounting for surface area of the sensor. The optimized sensors were characterized 3 different ways: using commercially available reference and counter electrodes, using printed reference and counter electrodes, and using a printed reference electrode with no counter electrode. Sensors intended for measuring sweat must be selective in the presence of sweat constituents. Thus, in addition to traditional characterization in pH 7.0 buffer, we characterized sensor performance in solutions intended to approximate sweat. Sensor performance in pH 7.0 buffer solution was not reflective of sensor performance in artificial sweat, indicating that further characterization is necessary between sensor measurement in pH 7.0 buffer and on-body measurements. Furthermore, we performed enzyme activity measurements and sensor measurements concurrently in five different salts individually, finding that while NH_4_Cl and MgCl_2_ do not affect enzyme activity or sensor performance in physiologically relevant ranges of salt concentration, NaCl concentration or KCl concentration decreases enzyme activity and sensor current. On the other hand, CaCl_2_ induced a nonlinear change in sensor performance and enzyme activity with increasing salt concentration.

## Introduction

Since the 1940s, lactate has been a metabolite of interest in bodily fluids because it has been shown to indicate a transition from aerobic to anaerobic metabolism^1–3^, and has been identified as a potential indicator for pressure ischemia^4^, panic disorder^5^, and cystic fibrosis^6,7^. In recent years, personalized and mobile, wearable health monitoring have become increasingly popular^8–11^. In particular, sweat promises to be a non-invasive way to monitor metabolites^7,12^. Sweat sensors provide a unique platform for non-invasive, continuous, wearable health monitoring. The advent of immobilizing enzymes enabled the development of enzymatic lactate sensors^13,14^. Since then, lactate sensors have been a popular area of study^2,3,15–18^, with some recent studies utilizing high-throughput fabrication methods, such as printing^2,15^.

An ideal lactate sensor will have high sensitivity and large linear range. In literature, the relevant range of lactate detection is up to 25 or 30 mM for sporting applications, especially in determining the transition between aerobic and anaerobic metabolism^19–33^. On the other hand, for clinical applications such as panic disorder, cystic fibrosis, and pressure ischemia, a linear range up to 50 mM is desired^5,7,34,35^.

Amperometric enzymatic sensors can be made at relatively low cost with a high sensitivity, making them ideal for commercial scale production, especially since the limited lifetime of enzymes dictates that enzymes are most appropriate for inexpensive disposable sensor applications. An amperometric lactate sensor is composed of 3 electrodes including a working electrode (WE), reference electrode (RE), and counter electrode (CE). A 2-electrode setup is occasionally chosen for ease of production, in which the counter and reference electrodes are combined into one electrode. Reference electrodes are typically composed of Ag/AgCl due to its ability to maintain a constant potential in varying ionic environments in addition to its ease of processing^36^. Counter electrodes and working electrode transducers are most commonly made of carbon, but may also be composed of gold, platinum and other inert metals which can be used to counter redox reactions^37,38^. The sensing layers on the working electrode transducer are fabricated using the enzyme lactate oxidase (LOX) immobilized in a polymer network, typically chitosan. LOX must be immobilized to prevent efflux of enzyme into the surrounding environment. Most sensors also employ a mediator in the composition of the working electrode, such as Prussian Blue^39,40^ or tetrathiafulvalene (TTF)^41–43^. Mediators function to lower the operation voltage of the sensor. LOX has an oxidation voltage arount 0.6 V^44,45^ when measured with an Ag/AgCl reference, at which other species in sweat such as uric acid and ascorbic acid also oxidize^46,47^. Oxidation of multiple species at the sensor surface introduces interference in the sensor and reduces its selectivity. Thus, a mediator with a low oxidation voltage is vital to fabricating a selective lactate sensor.

Printing addresses many of the unique needs of enzymatic sensors. Components of all 3 electrodes are solution-processable. Printing can be done on a variety of disposable substrates. Printing is also a high-throughput additive process with the potential to reduce cost of production, leading to inexpensive products which are necessary in the case of disposable sensors such as enzymatic sensors. Sensors designed to wear during a workout must be able to reliably continuously monitor a metabolite while remaining comfortable. The close contact afforded by flexible substrates ensures that sensors come in close contact with sweat as it is formed, giving the potential for highly accurate measurements. Solution-printing is compatible with flexible substrates with form factors closely matching that of skin. Conventional electronic fabrication technology does not allow for this flexibility.

This work utilizes TTF as a mediator due to its low oxidation voltage, high sensitivity, and compatibility with dermal applications. TTF is a small molecule usually used in organic semiconductors and organic charge transfer complexes and is known for its crystalline nature^48,49^. The sensors reported here are fabricated with ink-jet printed gold electrodes as the WE and CE and screen-printed Ag/AgCl as the RE to produce fully additively manufactured lactate sensors. By optimizing mediating and enzyme layer components of the working electrode, we report linear range up to 24 mM lactate and sensitivity up to 4.8 *μ*A/mM which normalizes to 68 *μ*A*cm^−2^/mM when accounting for surface area, which is suitable for sporting applications in both sensitivity and linear range. Optimized sensors were characterized in both 3-electrode and 2-electrode setups, indicating that while 2-electrodes are easier to fabricate and integrate with external electronics, 3-electrode setups offer better performance in more physiologically relevant ranges. We produced sensors which showed 97% current retention when operated continuously for an hour *in vitro* to mimic a typical workout.

Salts are well-known to affect enzyme activity^50^, though the effect of salt on enzymatic sensor performance is not yet well known. This work endeavored to characterize the effects of 5 different salts in ranges of physiologically relevant concentrations, though there are hundreds of constituents of sweat which have not yet been characterized for their effect on sensor performance^51^. This work incorporated salts and various solvents to mimic characteristics of sweat in a controlled lab environment. The ability to mimic *in vivo* characterization would allow us to produce a calibration curve that mimics sensor performance on the body in real sweat conditions. This is important to interpret lactate sensor data in real time if lactate sensors are ever to be used commercially. Ultimately, while sensors may perform well in a typical *in vitro* testing, this performance is not indicative of how sensors will perform in the presence of salts without a pH buffer present. This work investigates five chloride salts that are contained in sweat and considered primary electrolytes, though sweat has many more components which are not investigated in this work. Each salt affects sensor performance and enzyme activity differently. Until each component of sweat is investigated and accounted for alongside a lactate sensor, current lactate sensors can at best be labeled indicators of aerobic or anaerobic metabolism, but cannot give accurate lactate sweat concentrations.

## Results

### Sensor operation

**Figure 1:**
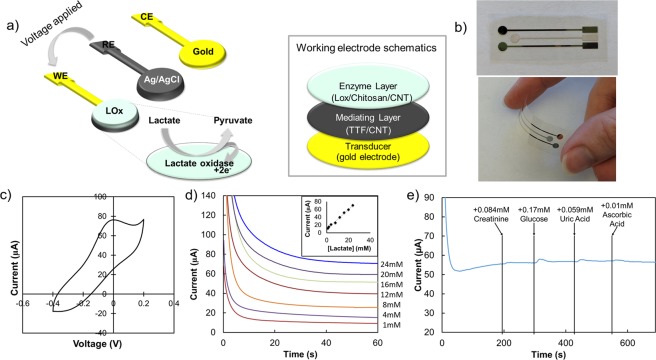
(**a**) A schematic of a 3-electrode lactate sensor indicating chemical reactions occurring on the working electrode including a schematic of the working electrode sensing layers on the right of (**a**,**b**) images of printed gold and Ag/AgCl electrodes prior to deposition of working electrode layers, with electrodes of 3 mm diameter, pictured straight and flexed; (**c**) Cyclic voltammetry graph of a TTF-mediated lactate sensor vs. Ag/AgCl reference; (**d**) Chronoamperometry of a lactate sensor with inset of steady-state points graphed to plot sensitivity; (**e**) Standard interference plot of a lactate sensor.

An amperometric lactate sensor is composed of 3 electrodes including a working electrode (WE), reference electrode (RE), and counter electrode (CE), as shown in Fig. 1a. Figure 1b shows these electrodes printed on plastic for a wearable form factor. Voltage is controlled between the RE and WE, and the surface of the WE is where reactions occur which release electrons that are read out as current. The RE is composed of printed Ag/AgCl, and the WE and CE are made of printed gold. The WE employs the enzyme lactate oxidase (LOX) as the sensing mechanism immobilized by the polymer chitosan with carbon nanotubes (CNTs). LOX has a high oxidation voltage around 0.6 V (vs. Ag/AgCl)^44,45^ at which other components in sweat oxidize and can introduce interference^46,47^, thus a mediator is used to lower the operation voltage. The mediator oxidizes at a lower voltage, spurring reactions between lactate and LOX. The mediating layer, composed of a mediator and CNTs, is placed below the enzyme layer. A mediator such as tetrathiafulvalene (TTF) has an oxidation voltage between 0–0.2 V when measured with an Ag/AgCl reference electrode, lower than that of LOX and other species found in sweat, preventing interference through oxidation. There are numerous mediators available for facilitating LOX reactions, though TTF has been shown to give high sensitivities and be compatible with dermal application^2^.

There are 3 main figures of merit for lactate sensors: operating voltage, sensitivity, and selectivity. The operating voltage is measured using cyclic votammetry (CV) such as the CV plot in Fig. 1c which was measured using an Ag/AgCl reference. The first oxidation peak indicates the oxidation of TTF to TTF+ which enables all of the reactions in the sensor. In Fig. 1c, the first TTF oxidation peak occurs at 0 V. Reduction peaks do not appear when performing CV on a lactate sensor due to the reversibility of the reactions within the sensor. Since oxidized TTF is reduced by LOX, it is not reduced by changing the voltage applied to the sensor and thus does not produce a reduction peak in the CV graph. For lactate sensors, lower magnitude operation voltages are ideal since high operation voltage can lead to interference with other species present in sweat.

The sensitivity of a lactate sensor is extracted from its chronoamperometry graph, such as that in Fig. 1d. Chronoamperometry is the measure of current in the sensor over time in increasing concentrations of lactate. The steady state current for each lactate concentration is plotted against lactate concentration to extract a linear fit and thus a sensitivity, as shown in the inset of Fig. 1d where the current recorded at 60 s is plotted against the corresponding lactate concentration.

The selectivity of a sensor is determined from its interference plot, as illustrated in Fig. 1e. In an interference plot, the current of a sensor in a single concentration of lactate is measured as other sweat metabolites are added. An ideal sensor shows minimal current change in the presence of each species introduced, as does the sensor in Fig. 1e. Uric acid and ascorbic acid are included due to their low oxidation voltages^46,47^ and show that the sensor operation voltage is low enough to prevent interference, while glucose and creatinine both undergo enzymatic reactions^52,53^ and show the selectivity of the enzyme itself.

The reactions that occur within the working electrode are shown in Eqs (1), (2) and (3):

1 $$Lactate+LOX(FAD)\leftrightarrow Pyruvate+LOX(FAD{H}_{2})$$

2 $$LOX(FAD{H}_{2})+2TT{F}^{+}\leftrightarrow LOX(FAD)+2TTF+2{H}^{+}$$

3 $$2TTF\leftrightarrow 2TT{F}^{+}+2{e}^{-}$$

where LOX refers to lactate oxidase enzyme, FAD refers to Flavin adenine dinucleotide of lactate oxidase, and TTF refers to tetrathiafulvalene. Equations (1), (2), and (3) occur simultaneously once TTF is oxidized. Lactate reacts with LOX to produce pyruvate and reduce the FAD of LOX. The reduced version of LOX reacts with oxidized TTF to oxidize LOX and reduce TTF. TTF is oxidized to produce electrons, which are read out as current.

### Sensor optimization

For sporting applications, sensors must achieve high sensitivity, with a linear range up to 30 mM lactate. Sensors with this linear range are best suited to sense the transition from aerobic to anaerobic metabolism. In order to achieve this, we optimized sensors based on sensitivity and linear range by changing the concentration of the TTF/CNT dispersion, the amounts of chitosan, CNT, and enzyme in the enzyme layer. Each of these parameters was optimized independently.

**Figure 2:**
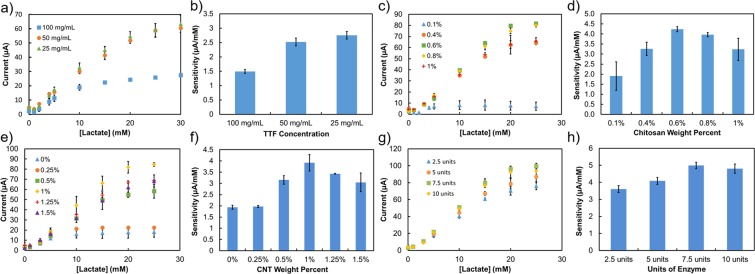
Characterization of mediating and enzyme layers: (**a**) showing current and linear range and (**b**) showing sensitivity comparison for each concentration of TTF/CNT suspension; (**c**) showing current and linear range and (**d**) showing sensitivity comparison for each weight percent of chitosan; (**e**) showing current and linear range and (**f**) showing sensitivity comparison for each weight percent of CNT; (**g**) showing current and linear range and (**h**) showing sensitivity comparison for each amount of units of enzyme. Error bars indicate standard deviation using at least 3 samples.

The mediating layer was optimized by changing the concentration of the TTF/CNT dispersion deposited on the working electrode surface. Because TTF is an organic semiconductor, conductive CNTs are employed to facilitate electron transfer. Since TTF is a small molecule, it crystallizes when deposited from solution. TTF forms smaller crystals when deposited from lower concentrations. With larger surface area to volume ratio, smaller crystals can contact the enzyme layer in more areas. 3 different concentrations of TTF/CNT dispersion were investigated, listed in Fig. 2a,b by the TTF concentration as 100 mg/mL, 50 mg/mL, and 25 mg/mL. The other three parameters were held at 1% chitosan, 1% CNTs, and 12 units of enzyme. The concentrations of CNT in the dispersion changed corresponding to the concentration of TTF, such that for 100 mg/mL TTF the concentration of CNT was 5 mg/mL, and when the TTF concentration was diluted the CNT concentration was correspondingly diluted. Thus for 50 mg/mL TTF the CNT concentration was 2.5 mg/mL and for 25 mg/mL TTF, the CNT concentration was 1.25 mg/mL. For each of these concentrations of TTF, the volume of dispersion deposited was changed such that the same solid content was deposited for each concentration, from 3 *μ*L for the highest concentration to 12 *μ*L for the lowest. Figure 2a,b shows the effect of TTF/CNT concentration on the linear range (2a) and sensitivity (2b) of the sensor. The linear range and sensitivity increase from up to 10 mM to up to 15 or 20 mM lactate and from 1.5 to 2.7 respectively when TTF/CNT concentration decreases from 100 mg/mL to 25 mg/mL. This occurs because regardless of the concentration, the dispersion is deposited in 3 *μ*L drops, one drop for the highest concentration to four drops for the lowest. Since these drops contain acetone and ethanol as solvents, which are both low boiling point and high vapor pressure, the solvent evaporates before large crystals of TTF are allowed to form. However, for higher concentrations the crystals are larger and contain more impurities than a lower concentration of the same volume^54^. Thus the crystals of TTF formed from lower concentrations are smaller and cover a larger proportion of the sensor surface, facilitating closer contact between the TTF and the LOX molecules. 25 mg/mL TTF was determined to give sufficient sensor performance for sporting applications, so 25 mg/mL TTF was used in further optimization tests.

The enzyme layer was optimized in 3 steps: chitosan weight percent, CNT weight percent, and enzyme loading, shown in Fig. 2c–h. The amount of chitosan was optimized by varying the weight percent included in the enzyme layer. The other three parameters were held constant at 25 mg/mL TTF, 1% CNT, and 12 units of enzyme. Chitosan acts as an immobilizer and prevents efflux of enzyme into the surrounding environment, but chitosan is also a resistive polymer. This means that if too little chitosan is included in the layer formulation, the enzyme will not be properly immobilized. Improper immobilization results in lower linear range and sensitivity, as can be seen in Fig. 2c,d, respectively, for 0.1% chitosan. When there is not enough chitosan to immobilize LOX, the linear range barely reaches 5 mM lactate. By comparison, when the enzyme is properly immobilized the linear range is up to 20 mM lactate. On the other hand, if too much chitosan is included, it can block the access between lactate and LOX and provide resistive losses. This results in lower currents and thus lower sensitivity, as can be seen in Fig. 2c,d, respectively, for 1% chitosan. From Fig. 2c,d, the chitosan weight percent that gave the highest sensitivity and highest currents was 0.6%, which was used thereafter for optimizing the next steps of the enzyme layer.

The amount of CNT was optimized by varying the weight percent included in the enzyme layer. The other three parameters were held constant at 25 mg/mL TTF, 0.6% chitosan, and 12 units of enzyme. CNTs provide conductive pathways to aid in charge transfer efficiency between the enzyme and solution. If there are not enough CNTs in the sensor, charges will have more difficulty transferring between spatially separated ions. The result is lower currents and thus lower sensitivity, as can be seen in Fig. 2e,f, respectively, for 0% and 0.25% CNTs. The linear range in both cases barely reaches 5 mM lactate, as it is visible in Fig. 2e, indicating that not all of the enzyme included in the sensor is acting to its full reactivity. On the other hand, including too much CNT in the sensor can crowd LOX molecules, potentially blocking them from reacting with Lactate. Too much CNTs can result in lower current and thus lower sensitivity, as shown in Fig. 2e,f, respectively, for 1.5% CNT. From Fig. 2e,f, 1% CNT gave the highest sensitivity and highest currents.

The enzyme loading was optimized by varying the units of enzyme included in the enzyme layer. The other three parameters were held constant at 25 mg/mL TTF, 0.6% chitosan, and 1% CNT. Lower enzyme loading results in resistive losses from too much chitosan, while higher enzyme loading can lead to efflux of enzyme into the surrounding environment. From Fig. 2g,h, 7.5 units of enzyme gives the highest current and sensitivity. The resulting sensor has a linear range up to 20 mM lactate but is sensitive up to 25 mM lactate, as shown in Fig. 2g, and has a high sensitivity as shown in Fig. 2h.

Through optimization of the mediating and enzyme layers of a lactate sensor, the sensor performance was improved from 1.5 *μ*A/mM or 21 *μ*A*cm^−2^/mM sensitivity with a linear range up to 10 mM lactate to a more physiologically relevant linear range up to 24 mM lactate with sensitivity of 4.8 *μ*A/mM or 68 *μ*A*cm^−2^/mM. Device-to-device reproducibility in performance is shown in Supplementary Fig. S1, wherein the calibration curves for 4 different sensors are overlaid on the same graph showing close agreement for corresponding concentrations of lactate. The resulting optimized sensors show sufficient performance for sporting applications of lactate sensing.

### Optimized sensor characterization

**Figure 3:**
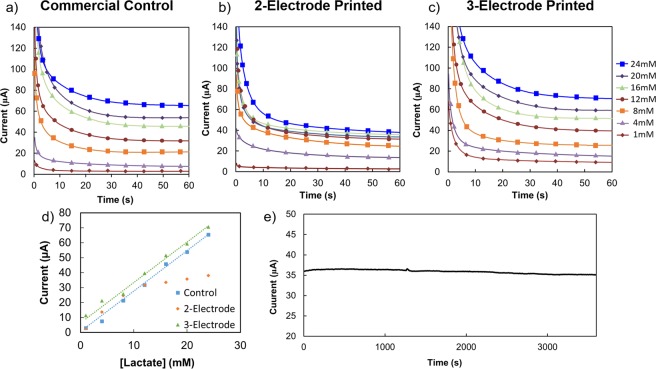
Chronoamperometry in three different configurations including (**a**) commercial 3-electrode control configuration, (**b**) 2-electrode printed configuration, and (**c**) 3-electrode printed configuration, with steady state currents extracted to sensitivity graph (**d**), and (**e**) sensor performance in one lactate concentration when operated continuously for one hour.

Printed working electrodes were optimized using a commercially available Ag/AgCl reference and a commercially available platinum counter electrode, referred to in Fig. 3 as the Commercial Control. The resulting optimized sensor was characterized using chronoamperometry in 3 different sensor setups including the Commercial Control. The chronoamperometry in the Commercial Control configuration for concentrations of lactate of 1 mM, 4 mM, 8 mM, 12 mM, 16 mM, 20 mM, and 24 mM is shown in Fig. 3a. These concentrations of lactate mimic physiological levels of lactate in sweat for sporting conditions. The printed working electrode was also tested using a printed reference electrode in a 2-electrode printed configuration with chronoamperometry shown in Fig. 3b for the same lactate concentrations, as well as a 3-electrode printed configuration with chronoamperometry shown in Fig. 3c using printed counter and working electrodes with the same lactate concentrations. Figure 3a,c look very similar, indicating that the printed reference and counter electrodes work just as well as the commercially available reference and counter electrodes. However, the 2-electrode setup in Fig. 3b exhibits clearly lower currents. The effects of the 2-electrode setup are even clearer in Fig. 3d, the sensitivity plots of each electrode configuration. The currents at 60 s for Fig. 3a,b, and c were plotted against lactate concentration to produce the sensitivity plot shown in Fig. 3d. While the commercial and printed 3-electrode configurations have the same sensitivity, offset by a small but constant current, the 2-electrode configuration does not have the same linear range as the other two. The 2-electrode configuration relies on the Ag/AgCl electrode to perform the function of both reference and counter electrodes. The reference electrode must maintain a stable potential in varying ionic environments, while the counter electrode works to counter the redox reactions that occur on the surface of the working electrode by allowing current to pass between the counter and working electrodes. It is challenging to maintain a constant potential while supplying current to the working electrode, thus 2-electrode configurations are expected to show lower currents and linear ranges than their 3-electrode counterparts^37^. This is demonstrated in Fig. 3. The 3-electrode configurations, however, exhibit linear range and sensitivity sufficient for sporting applications. Furthermore, when operated continuously for an hour in a single concentration of lactate as shown in Fig. 3e, the sensor exhibits 97% current retention, indicating this sensor could be useful for continuously monitoring an exercise session. The *in vitro* results in Fig. 3 indicate a promising sensor for determining the transition between aerobic and anaerobic metabolism. The current stability of the sensor is further studied by measuring a single sensor repeatedly over the course of 4 hours in varying concentrations of salts. The results are shown in Supplementary Fig. S2, where the sensors were measured in increasing concentrations of lactate in water, then allowed to rest in deionized water before measuring again in lactate in water with added salt. In the case of MgCl_2_, which has little interference with sensor performance, the current in the sensor at each concentration of lactate does not vary. For example, at 15 mM lactate, the current is 17.7 *μ*A + /−0.5 *μ*A.

**Figure 4:**
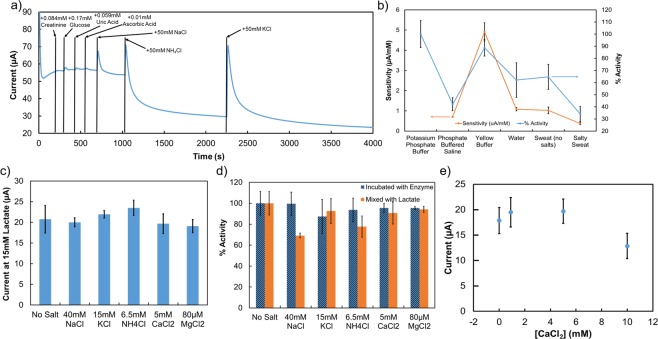
Sensor performance in sweat-like conditions including: (**a**) traditional interference species plus salts; (**b**) sensor sensitivity and relative enzyme activity in varying solutions; (**c**) current in a lactate sensor at 15 mM lactate with physiological concentrations of five different salts; (**d**) enzyme activity two ways in the presence of the same five salts; (**e**) sensor current at 15 mM lactate for four different concentrations of one salt. Error bars indicate standard deviation using at least 3 samples.

The last figure of merit to address is selectivity. Our sensor was characterized for selectivity in the traditional manner by continuously operating the sensor in 15 mM lactate and adding potentially interfering species one at a time, as shown in Fig. 4a. This work employed physiologically average concentrations of the standard interfering species for lactate: 0.084 mM creatinine, 0.17 mM glucose, 0.059 mM uric acid, and 0.01 mM ascorbic acid. Traditionally, creatinine, glucose, uric acid, and ascorbic acid are included in interference studies for lactate for two different reasons. Creatinine and glucose both undergo reactions with enzymes and are included to test the selectivity of the LOX enzyme for lactate. Uric acid and ascorbic acid both have low oxidation voltages and are included to test that the operation voltage of the sensor is low enough to prevent interference from oxidation. From Fig. 4a, these 4 species introduced minimal variation in sensor current, indicating no interference. In addition, this work acknowledges that salts are a major component of sweat^51^, and included 3 extra salts which are major components of sweat: NaCl, NH_4_Cl, and KCl. Sodium, ammonium, potassium, and chloride are all main components of sweat and thus provide a rigorous first step toward real sweat conditions. From Fig. 4a, each of these 3 chloride salts in 50 mM concentrations introduced major changes in current to the sensor, and thus interfere with sensor operation. For sodium, 50 mM is a physiologically relevant concentration, but for ammonium and potassium, physiologically relevant concentrations are much lower at 5 mM and 8 mM respectively. The interference observed in Fig. 4a led to a deeper exploration of interference between enzymes and salts and an attempt to approximate real sweat *in vitro*.

To further approximate *in vivo* conditions in comparison with typical *in vitro* characterization, a variety of solutions were characterized *in vitro* including various buffers typically used to characterize sensors as well as non-pH-buffered solutions. Sensor performance, as well as enzyme activity are shown in Fig. 4b. Enzyme activity is shown in blue with activity reported as a percentage relative to the standard potassium phosphate buffer used for enzyme assay as reported by Toyobo^55,56^. The scale for enzyme activity percentage is shown on the right of the graph. Sensitivity of corresponding sensors in solutions of lactate using each type of solution is shown in orange, with sensitivity scale shown on the left of the graph. The first solution listed is Potassium Phosphate Buffer, referring to the standard buffer used by Toyobo with a pH of 7.5, followed by Phosphate Buffered Saline from Glibco, a pH 7.4 buffer that includes sodium chloride in the formulation and is often used in sensor characterization. Yellow Buffer refers to Fisher’s phosphate buffer with pH 7.0 which includes potassium hydroxide in the formulation. This is the solution which was used in this work for optimization and characterization of sensors. Water refers to deionized water. Sweat with no salts and Salty Sweat refer to house-made artificial sweat recipes using physiologically average amounts of the previously tested interference species. 84 *μ*M creatinine, 0.17 mM glucose, 59 *μ*M uric acid, and 10 *μ*M ascorbic acid were dissolved in deionized water for Sweat (No Salts), while Salty Sweat includes these as well as 50 mM NaCl, 5 mM NH_4_Cl, and 8 mM KCl. As indicated by the first 700 s of Fig. 4a, Sweat with no salts produces similar results in both sensitivity and enzyme activity as plain deionized water. The traditionally measured interfering species for lactate have very little effect on our sensor’s performance.

Five different chloride salts were investigated for their effects on enzyme activity and sensor performance: NaCl, NH_4_Cl, KCl, CaCl_2_, and MgCl_2_, each of which is a constituent in sweat. The effect of nearly physiologically average concentrations of each of these salts on sensor current at 15 mM lactate is shown in Fig. 4c. While some salts give lower currents at physiologically average concentrations (NaCl, CaCl_2_, and MgCl_2_), others give higher currents (NH_4_Cl and KCl). To help explain why these salts affect sensor current, the same salt concentrations were used in Fig. 4d when measuring enzyme activity. In Fig. 4d, two different methods were employed for measuring enzyme activity in order to find one method which might predict sensor performance. The standard method for measuring enzyme activity in the presence of other species is shown in blue. This method involves incubating the enzyme with the determined concentration of salt for an hour prior to dilution for assay. In orange is a method from this work of mixing the salt with the lactate in the assay mixture, to mimic the changes that a salt may bring about to the redox reactions on a sensor. For some salts such as NaCl and NH_4_Cl, incubation was a more accurate indicator of a salt’s effect on sensor performance, indicating that salt affected the enzyme most within the sensor. Because the enzyme is the sensing mechanism, changes in enzyme activity will change a sensor’s current output, even in the presence of the same concentration of lactate. The sensor performance in the presence of the other salts, KCl, CaCl_2_, and MgCl_2_, was better indicated by mixing salt with the assay mixture, indicating that the salt affects ionic interactions surrounding a sensor more than the enzyme itself. This form of interference still affects sensor current because the ionic interactions prevent or facilitate lactate from reaching the enzyme to react and produce current in the sensor. The changes in sensor current corresponding from changes in enzyme activity are more clearly visible in Supplementary Fig. S2, in which sensors are investigated with a range of salt concentrations to determine their effects on sensitivity of a sensor as well as enzyme activity.

Even for a single salt, the effect on the sensor is highly dependent upon salt concentration, as seen in Fig. 4e, in which the current of a sensor in 15 mM lactate is plotted for 4 different concentrations of CaCl_2_. The sensor response to increasing concentrations of CaCl_2_ is nonlinear.

We have further investigated NaCl, NH_4_Cl, KCl, CaCl_2_, and MgCl_2_ for their effect on enzyme activity and sensor performance. The results of this study are shown in Supplementary Fig. S2, where sensitivity is compared to enzyme activity using the traditional method of incubating the enzyme with salt prior to dilution for assay, as well as a novel enzyme activity method of mixing salt with the assay mixture. For NaCl and KCl, increasing the concentration of salt reduces sensitivity of sensors, following the enzyme activity trend of salt mixed with assay mixture. For NH_4_Cl and MgCl_2_, increasing the concentration of salt within physiological ranges seems to have no particular effect on sensor sensitivity or enzyme activity. Increasing CaCl_2_ concentration has a nonlinear effect on sensor performance, increasing sensitivity between 0 mM and 0.9 mM CaCl_2_ and decreasing sensitivity between 0.9 mM and 10 mM CaCL_2_, which follows the trend for incubated enzyme activity when increasing and mixed enzyme activity when decreasing.

The use of salts and non-buffered solutions was vital to determining more closely how sensors may perform *in vivo*, since sweat is a salty, non-buffered environment. Each salt affected sensor performance and enzyme activity differently, though the effect on enzyme activity indicates that salt is a major factor to take into consideration when developing enzymatic sensors.

## Discussion

An ideal, printed, flexible, wearable lactate sweat sensor holds many advantages over the current methods of measuring bodily lactate. Instead of monitoring blood, it non-invasively monitors sweat. It has the ability to monitor in real-time as opposed to collecting a sample for lab-scale spectroscopic testing. While the advantages of a wearable sensor are great, the accuracy of blood and spectroscopic sweat testing cannot necessarily be met by these sensors.

The sensitivity of lactate sensors in Fig. 4b very closely follows the trend for enzyme activity, which indicates that changing the lactate solution most affects the enzyme in an enzymatic sensor, not necessarily the other electrochemical reactions taking place in solution. Since the enzyme is the mode of sensing in enzymatic sensors, variations in activity are not ideal. To perform well, a sensor must react predictably to changing environments, ideally reacting selectively only to the constituent of interest.

While optimized sensors perform well in a pH-buffered *in vitro* environment, introduction of any variables that bring conditions closer to those of *in vivo* measurements drastically affects sensor performance and render previously acquired sensitivity graphs inaccurate for comparing with on-body measurements. To better mimic on-body environments and get closer to accurate *in vivo* performance, artificial sweat should be used to characterize sensors. However, enzyme activity is known to be highly dependent upon temperature, pH, and salt^50,55,56^, and while our study discusses five salts, there are hundreds of constituents of sweat^51^. In order to detect a transition between aerobic and anaerobic metabolism, a change in lactate in sweat of approximately 10 mM is expected. In our sensors, this change in lactate concentration corresponds to a change in current of approximately 10 *μ*A. When we consider salt interference as shown in Fig. 4c, the current in sensors at physiologically average concentrations of salt changes approximately 5 *μ*A. The change in current due to salt interference is lower than the change in current required to detect the threshold between aerobic and anaerobic metabolism. Therefore, these sensors may still function well as indicators for the purposes of determining the transition between aerobic and anaerobic metabolism, even taking into account the interference of salts. However, it is unlikely that enzymatic lactate sensors will give accurate numerical readings for continuous health monitoring. The effects of each of the constituents in sweat on enzymatic sensors are not well known. In order to achieve an accurate measurement of lactate, a systematic study of each constituent in sweat would need to be performed for its effect on enzymatic sensors. Any constituents found to affect sensor performance would need to be included in a multiplexed sensor configuration where its effects are back-calculated to determine an accurate numerical value for lactate in sweat. From our studies, we conclude that at least sodium, potassium, and calcium must be included in such a multiplexed patch, in addition to pH and temperature before on-body studies are performed.

## Methods

Chitosan and TTF were obtained from Sigma Aldrich. Lactate Oxidase was obtained from Toyobo. Carbon nanotubes were obtained from Carbon Solutions, Inc in the form of iP-Single Walled Carbon Nanotubes. Acetone, ethanol, and acetic acid were obtained from Sigma Aldrich. Yellow pH 7.0 buffer was obtained from Fisher Scientific.

Gold electrodes were printed using commercially available Harima Nanopaste(Au) NPG-J gold ink in a Dimatix inkjet printer at ambient conditions. Dimatix inkjet printers employ a piezo inkjet cartridge. When a waveform is applied, ink droplets are deposited on the substrate below. The desired pattern is drawn in software such as AutoCAD and converted to a format compatible with Dimatix software for waveform application. Printed gold electrodes are sintered at 250 °C for 50 minutes. Ag/AgCl was printed using Engineered Materials Systems, Inc. CI-4001 ink in a screen printer. During screen printing, a silk screen with a predetermined pattern is flooded with ink. The ink then gets pressed through the screen onto a substrate below. Printed Ag/AgCl electrodes are then baked at 110 °C in a vacuum oven for 2 hours. Electrodes were printed on PQA2 PEN 25 *μ*m thick. Printed electrodes were encapsulated using laser-cut Teflon tape. Printed electrodes had circular active areas of 3 mm diameter, resulting in an active area of 0.07068 cm^2^. This area was used to calculate sensitivity per area.

To make the mediating layer, carbon nanotubes were dispersed in ethanol in the specified concentration (1.25 mg/mL for optimized sensors) and sonified for 20 minutes at 40% amplitude using a Branson Digital Sonifier probe. TTF was dissolved in acetone at the corresponding concentration (25 mg/mL for optimized sensors). 400 *μ*L of TTF solution was added to 2 mL CNT dispersion, and the resulting solution was sonified for 20 minutes at 40% amplitude. The TTF/CNT dispersion was then deposited on the working electrode surface (4 drops of 3 *μ*L for optimized sensors).

To make the enzyme layer, chitosan was dissolved in 1% acetic acid in water (0.6% chitosan by weight for optimized sensors), and CNTs were added (1% by weight for optimized sensors). This mixture was sonified for 20 minutes at 40% amplitude. In a separate vial, Lactate Oxidase was measured out and dissolved in Fisher pH 7.0 buffer (for optimized sensors, 1500 U/mL). The lactate oxidase mixture was mixed 1:1 with the chitosan and CNT mixture and deposited on top of the mediating layer (1 drop of 10 *μ*L for optimized sensors). The sensors were then dried overnight in an Environmental Chamber at 35 °C.

Optimization results were measured using commercial control setup involving printed WE, commercially available Ag/AgCl RE, and commercially available Pt wire CE. Reference electrodes were obtained from Koslow. All optimization was performed in Fisher pH 7.0 buffer containing potassium phosphate monobasic, sodium hydroxide, and water. Sodium-L-lactate was obtained from Sigma Aldrich.

Uric acid, ascorbic acid, glucose, creatinine, sodium chloride, potassium chloride, ammonium chloride, magnesium chloride, and calcium chloride were all obtained from Sigma Aldrich.

Enzyme activity was performed using the procedure from Toyobo. Potassium phosphate buffer of pH 7.5 was made using potassium phosphate monobasic obtained from Sigma Aldrich, potassium phosphate dibasic obtained from Sigma Aldrich, and potassium hydroxide obtained from Sigma Aldrich. When comparing enzyme activity in various solutions, the potassium phosphate buffer of pH 7.5 in which the lactate was dissolved was exchanged for the various other solvents. Gibco Phosphate Buffered Saline was obtained from ThermoFisher Scientific. Enzyme was incubated with salt according to the procedure from Toyobo, salt was also mixed with the lactate solution prior to assay. Absorbance was measured using a Shimadzu UV-2600 UV-Vis Spectrometer. Activity was calculated using the method from Toyobo, then divided by calculated activity using the standard Toyobo procedure with no salt and no solvent substitutions to arrive at a percentage activity. Through this method, the reference activity is always 100%.

Error bars in figures are calculated standard deviation of between 3 and 50 samples.

## Supplementary information

Supplementary Information

## Data Availability

All data needed to evaluate the conclusions in the paper are present in the paper and/or the Supplementary Materials. Additional data related to this paper may be requested from the authors.
