# Supplementary material for: Printed, Flexible Lactate Sensors: Design Considerations Before Performing On-Body Measurements
Source: Sci Rep. 2019 Sep 23;9:13720. doi: 10.1038/s41598-019-49689-7 (PMC6757068; doi:10.1038/s41598-019-49689-7)
Supplement: Supplementary file 1 — Supplementary Information [file 41598_2019_49689_MOESM1_ESM.pdf]

# Printed, Flexible Lactate Sensors: Design Considerations Before Performing On-Body Measurements

Margaret E. Payne[1,+], Alla Zamarayeva[1,+], Veronika I. Pister[1], Natasha A. D. Yamamoto[1], and Ana Claudia Arias[1,*]

[1]University of California Berkeley, Electrical Engineering and Computer Science, Berkeley, CA, 94720, United States
[*]acarias@eecs.berkeley.edu
[+]These authors contributed equally to this work.

**Supplementary Information**

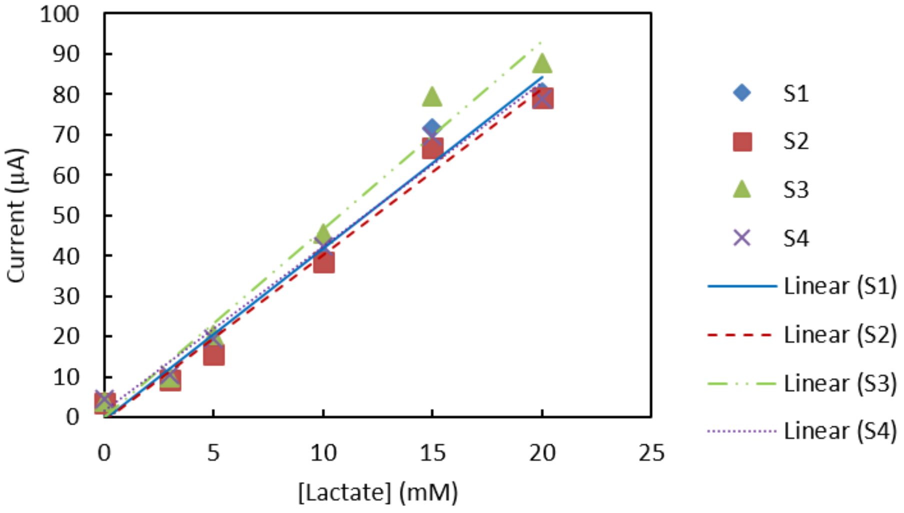


Supplementary Figure S1 shows reproducibility of sensor performance. Four sensors (S1, S2, S3, and S4) are shown with close agreement.

a)

NaCl incubated with enzyme

| | % Activity | St. Dev. (%) |
|---|---|---|
| 60mM | 95.6 | 17.7 |
| 40mM | 99.5 | 11.1 |
| 20mM | 110.4 | 8.2 |
| 10mM | 103.5 | 15.6 |

NaCl mixed with lactate

| | % Activity | St. Dev. (%) |
|---|---|---|
| 60mM | 59.5 | 3.3 |
| 40mM | 69.1 | 2.3 |
| 20mM | 81.3 | 3.7 |
| 10mM | 92.9 | 3.1 |

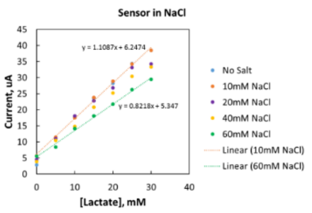


b)

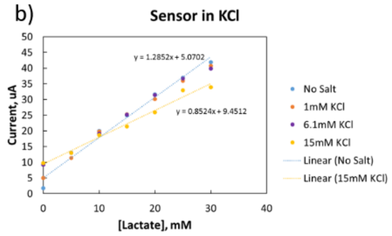


KCl incubated with enzyme

| | % Activity | St. Dev. (%) |
|---|---|---|
| 15mM | 87.4 | 16.2 |
| 6.1mM | 95.0 | 3.1 |
| 1mM | 116.6 | 2.1 |

KCl mixed with lactate

| | % Activity | St. Dev. (%) |
|---|---|---|
| 15mM | 92.6 | 11.9 |
| 6.1mM | 96.1 | 0.7 |
| 1mm | 103.9 | 2.0 |

c)

NH4Cl incubated with enzyme

| | % Activity | St. Dev. (%) |
|---|---|---|
| 10mM | 97.8 | 2.0 |
| 6.5mM | 93.7 | 11.1 |
| 3.3mM | 87.6 | 3.6 |
| 0.5mM | 89.9 | 2.5 |

NH4Cl mixed with lactate

| | % Activity | St. Dev. (%) |
|---|---|---|
| 10mM | 89.2 | 5.0 |
| 6.5mM | 77.6 | 10.2 |
| 3.3mM | 82.7 | 5.2 |
| 0.5mM | 105.6 | 6.3 |

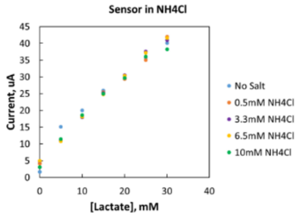


d)

MgCl2 incubated with enzyme

| | % Activity | St. Dev. (%) |
|---|---|---|
| 800uM | 92.9 | 3.4 |
| 80uM | 95.5 | 1.4 |
| 8uM | 94.9 | 1.1 |

MgCl2 mixed with lactate

| | % Activity | St. Dev. (%) |
|---|---|---|
| 800uM | 98.4 | 1.4 |
| 80uM | 94.2 | 2.7 |
| 8uM | 94.1 | 5.4 |

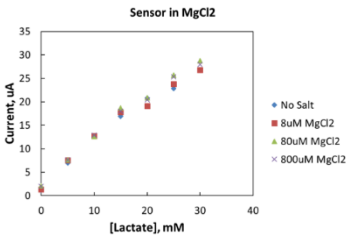


e)

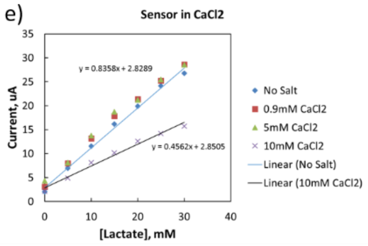


CaCl2 incubated with enzyme

| | % Activity | St. Dev. (%) |
|---|---|---|
| 10mM | 95.6 | 3.2 |
| 5mM | 95.5 | 4.4 |
| 0.9mM | 104.5 | 1.0 |

CaCl2 mixed with lactate

| | % Activity | St. Dev. (%) |
|---|---|---|
| 10mM | 78.5 | 14.7 |
| 5mM | 90.7 | 10.4 |
| 0.9mM | 92.3 | 2.3 |

Supplementary Figure S2 shows enzyme activity and single demonstrative sensor performance in a) NaCl, b) $NH_4Cl$, c) KCl, d) $CaCl_2$, and e) $MgCl_2$. For each salt, enzyme activity is shown in two tables: enzyme activity when the salt is incubated with the enzyme and enzyme activity when salt is mixed in

lactate assay solution. This demonstrates how drastically enzyme activity and sensor performance changes as salt concentration changes.
